# Polyglutamine Aggregate Structure *In Vitro* and *In Vivo;* New Avenues for Coherent Anti-Stokes Raman Scattering Microscopy

**DOI:** 10.1371/journal.pone.0040536

**Published:** 2012-07-20

**Authors:** Nicolas M. Perney, Lucy Braddick, Martin Jurna, Erik T. Garbacik, Herman L. Offerhaus, Louise C. Serpell, Ewan Blanch, Lindy Holden-Dye, William S. Brocklesby, Tracy Melvin

**Affiliations:** 1 Optoelectronics Research Centre, University of Southampton, Highfield, Hampshire, United Kingdom; 2 MESA+ Institute for Nanotechnology, University of Twente, Enschede, The Netherlands; 3 School of Life Sciences, University of Sussex, Falmer, Sussex, United Kingdom; 4 Faculty of Life Sciences and Manchester Interdisciplinary Biocentre, The University of Manchester, Manchester, United Kingdom; 5 School of Biological Sciences, University of Southampton, Highfield, Hampshire, United Kingdom; Dalhousie University, Canada

## Abstract

Coherent anti-Stokes Raman scattering (CARS) microscopy is applied for the first time for the evaluation of the protein secondary structure of polyglutamine (polyQ) aggregates *in vivo*. Our approach demonstrates the potential for translating information about protein structure that has been obtained *in vitro* by X-ray diffraction into a microscopy technique that allows the same protein structure to be detected *in vivo*. For these studies, fibres of polyQ containing peptides (D_2_Q_15_K_2_) were assembled *in vitro* and examined by electron microscopy and X-ray diffraction methods; the fibril structure was shown to be cross β-sheet. The same polyQ fibres were evaluated by Raman spectroscopy and this further confirmed the β-sheet structure, but indicated that the structure is highly rigid, as indicated by the strong Amide I signal at 1659 cm^−1^. CARS spectra were simulated using the Raman spectrum taking into account potential non-resonant contributions, providing evidence that the Amide I signal remains strong, but slightly shifted to lower wavenumbers. Combined CARS (1657 cm^−1^) and multi-photon fluorescence microscopy of chimeric fusions of yellow fluorescent protein (YFP) with polyQ (Q40) expressed in the body wall muscle cells of *Caenorhabditis elegans* nematodes (1 day old adult hermaphrodites) revealed diffuse and foci patterns of Q40-YFP that were both fluorescent and exhibited stronger CARS (1657 cm^−1^) signals than in surrounding tissues at the resonance for the cross β-sheet polyQ *in vitro*.

## Introduction

Coherent anti-Stokes Raman scattering (CARS) microscopy can be applied in living cells and provides vibrational contrast at a speed that is orders of magnitude higher than conventional Raman microscopy [1], [2]. The majority of CARS studies on living systems so far, have focused on the investigation of lipids; examples of these include studies of lipid storage in *Caenorhabditis elegans* nematodes [3] and lipid vesicles inside HeLa cells [4]. Lipids are relatively easy molecules to probe by CARS due to the high densities within structures *in vivo* and the strong signal at 2845 cm^−1^ assigned to the C-H vibrational stretch. Other regions of the spectrum can give information about other important molecules; for example, imaging at 1090 cm^−1^ of phosphate groups of chromosomes within cells undergoing mitosis [5], and imaging in the spectral region from 675 cm^−1^ to 950 cm^−1^ to make a comparison between un-differentiated and differentiating murine embryonic stem cells [6].

Since the Raman spectral region from ∼1000 cm^−1^ to ∼1800 cm^−1^ contains bands that can be considered as ‘fingerprints’ for the structure of proteins, given a level of knowledge/reference data it is possible to gain an understanding of the structure of protein molecules directly from the Raman spectrum of the ‘pure’ protein [7]. Examples include Raman marker bands for α-helix (1650 and 1340 cm^−1^), β-sheet (1690, 1740 and 1240 cm^−1^) and disordered protein structures (1670 and 1220 cm^−1^). Like Raman spectroscopy, CARS methods have also been used to evaluate protein structure *in vitro*. Perhaps the most important study in this area is the polarization sensitive CARS spectroscopy of the amide I band of various purified proteins *in vitro,* a result of a collaboration between the groups of Chikishev and Greve [8]. Contributions of α-helix, β-sheet and random coil to the amide I and II regions of the spectrum was obtained from the measured CARS spectra. Following on from these studies the group of Greve and Otto reported a resonance CARS study of the purified red fluorescent protein, DsRed and also recorded the spectrum of green fluorescent protein, GFP [9]. The major CARS band in the amide I region for GFP was shown to be 1615 cm^−1^.

So far CARS images obtained within this spectral region have not been recorded with a view to identifying particular molecules. Even though images have been obtained by tuning the excitation lasers for molecular vibration modes assigned to amide I, II or II of peptide bonds, *i.e.* [10], [11] and the results were clearly a first, the results cannot be used to understand anything about the protein structure *in vivo*. The reason for this is that the CARS voxel sizes are not sufficiently small to allow detection of anything other than a mixture of biomolecules.

In CARS microscopy voxels of the order of a few hundred nanometres are interrogated, thus protein aggregates, with a size on the micron scale, are ideal candidates for evaluation using CARS methods. In the studies described in this paper we interrogate polyglutamine (polyQ) aggregates, that are implicated in Huntington’s disease [12]. This genetic disease is caused by a CAG/polyQ expansion from a non-pathological length (Q20–Q40) to a disease-associated length (>Q40). Above this Q40 threshold polyQ-containing peptides/proteins misfold, oligomerise and form amyloid-like fibrils inside cells, a process that has been modelled extensively in the test-tube, cell and animal models. The structural changes associated with the polyQ aggregation process and its complex interplay with other proteins that recognise abnormal proteins (molecular chaperones) has not been elucidated in living systems to date. For these first studies, the aggregation process of polyQ provides a very tractable system for study because (i) strong Raman signals should be obtained against the background due to the highly dense nature of the aggregates, (ii) the *in vivo* aggregates are on the micron scale and most importantly, a significant volume of published work demonstrates that EGFP/other-tagged polyQ peptides within living mammalian cells and in *C. elegans* follow an aggregation process [13], [14], [15], [16], [17], [18], [19]; a system that is well understood is crucial for the implementation of an imaging technique, such as CARS microscopy, in a new manner.

## Results and Discussion

**Figure 1:**
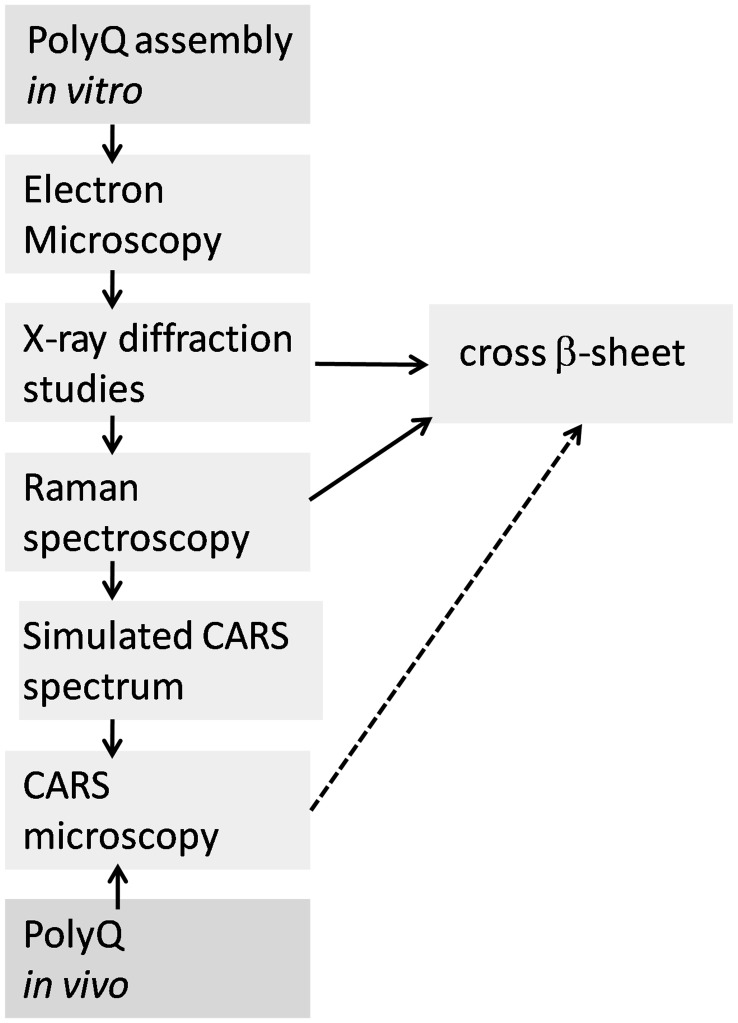
Schematic showing the bioanalytical tools required for evaluation of the polyQ structure *in vivo.*

This is a novel approach for protein structure evaluation *in vivo*, the various steps required are illustrated in figure 1. Our strategy for the detection and identification of polyQ aggregates *in vivo* by CARS microscopy relies upon effective characterisation of similar polyQ aggregates *in vitro*. The benchmark Raman spectral information for polyQ aggregates *in vitro* allows for the identification of protein structure *in vivo*, if common spectral signatures are observed.

**Figure 2:**
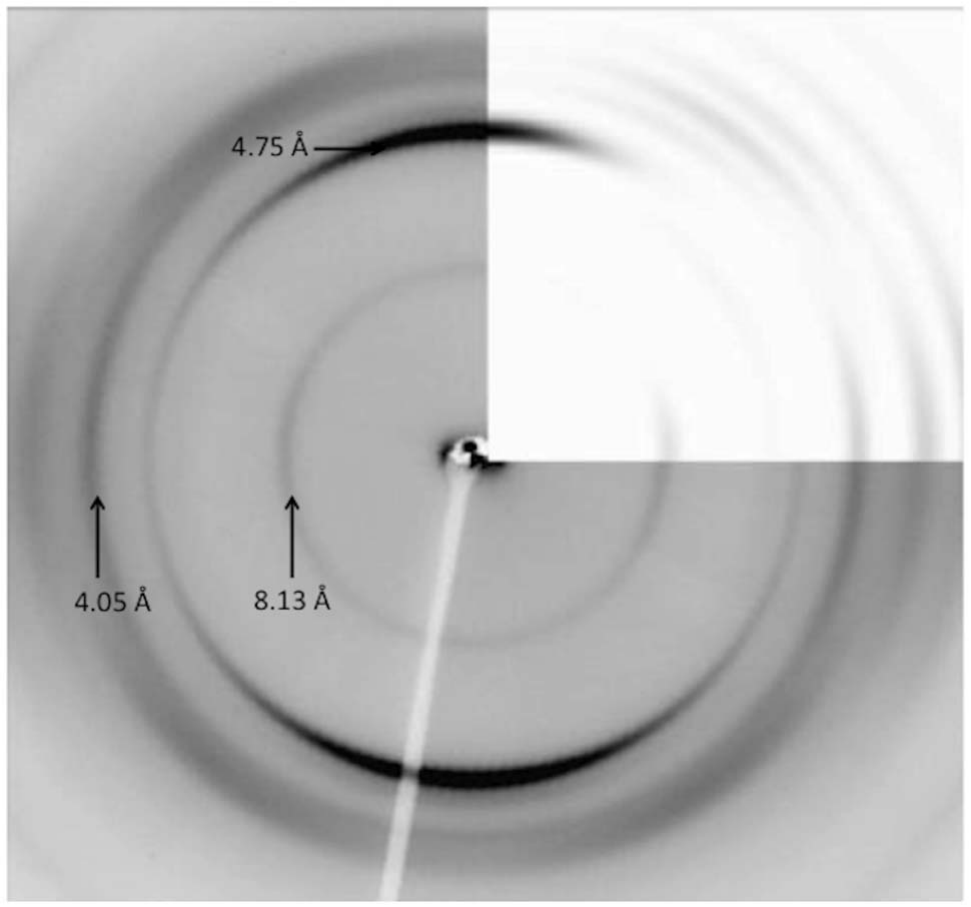
Experimental and calculated X-ray fibre diffraction pattern for fibres of the poly-L-glutamine peptide, D_2_Q_15_K_2_. The experimental X-ray fibre diffraction pattern has the fibre axis running vertical and the strong characteristic cross-β reflection at 4.75 Å and equatorial reflections at 8.13, 4.05, Å (see labels) 3.55 and 3.25 Å. Top right hand corner shows calculated diffraction pattern from coordinates [22] and shows an excellent match in signal positions and relative intensity.

The assembly of amyloid-like fibrils *in vitro* from a number of different polyQ peptide sequences as well as huntingtin exon 1 (htt exon 1) has previously been reported; the fibres have a particular morphology, often showing short, wide filaments [20]. X-ray fibre diffraction from peptides composed of htt exon 1 or from a polyQ peptide D_2_Q_15_K_2_ give patterns with characteristic cross-β reflections [21]. The interpretation was that a novel aggregate structure known as the “water-filled nanotube” is formed. This suggested structure is composed of a continuous β strand running around a cylinder of width approximately 63 Å [21]. Because the interpretation was controversial, reanalysis of the data collected by the Perutz team was performed again by Sikorski and Atkins to reveal a more conventional cross-β structure [22] in which hydrogen bonding was observed between the glutamine side chains. This reanalysis was performed in light of the observation that (i) the sample texture was not a true fibre and (ii) the nanotube model proposed was unlikely to fit available experimental data. The diffraction patterns expected from model structures of both the water filled nanotube and the cross β-sheet were calculated using a program, called CLEARER [23], [24]. The resulting data provided an excellent match between the experimental and calculated pattern for the cross-β type model, but not for the water filled nanotube model. Experimental studies using the same peptide sequence (D_2_Q_15_K_2_) as evaluated by Perutz *et al*. [21], were performed by ourselves; D_2_Q_15_K_2_ was incubated in water and the resulting short, stocky fibres detected and imaged by electron microscopy (Figure S1). These fibrils were partially aligned to form a fibre sample and X-ray fibre diffraction gave patterns, showing a cross-β like pattern (meridional at 4.75 Å and sharp equatorial at 8.13 Å with additional reflections not previously observed [21]). The diffraction patterns (obtained in these studies (Figure 2)) show very similar signal positions to those shown in the original diffraction pattern from Perutz *et al*., but with additional reflections arising from the improved organisation with the sample. Diffraction calculations using cell dimensions a = 9.5 Å, b = 16.6 Å, c = 6.95 Å α = β = γ = 90° and coordinates from the cross-β model proposed by Sikorski and Atkins confirmed an excellent match between signal positions from experimental and calculated patterns (Figure 2). The calculated pattern shows the sharp reflection on the equator at 8.13 Å which arises from the closely packed sheet spacing in the model structure [22].

**Figure 3:**
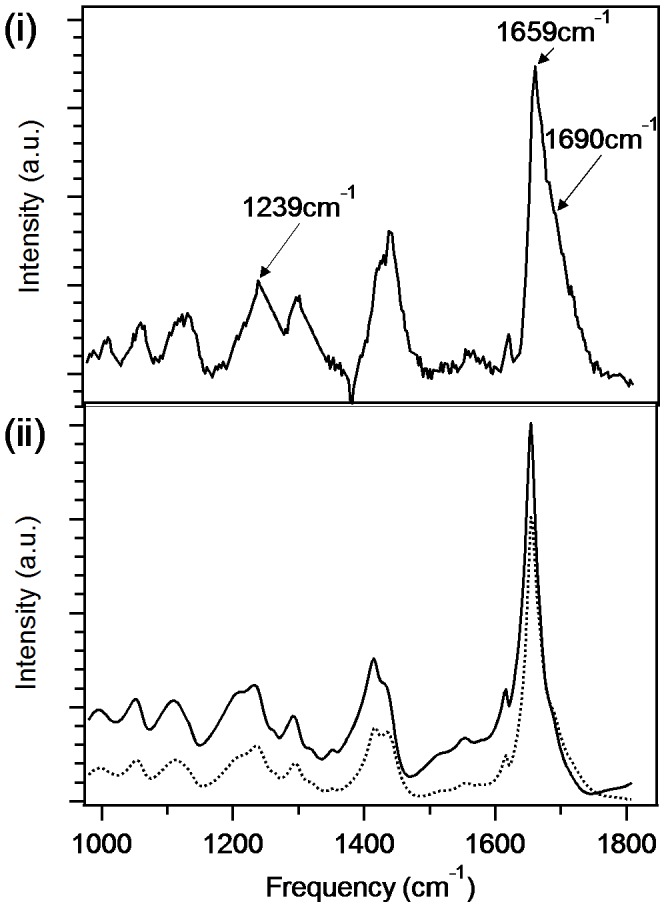
Raman and predicted M-CARS spectra of fibres of D_2_Q_15_K_2_. (i) Raman spectrum of the fibres of D_2_Q_15_K_2_ as evaluated by X-ray diffraction. (ii) Predicted M-CARS spectra (solid line corresponds to 50%, and dashed line corresponds to 20% non resonance contribution.)

It is unfortunate that purified proteins or peptides, already characterised by NMR or crystallography methods, have been rarely assessed by Raman spectroscopy methods. The same sample of fibres as used for the electron microscopy and X-ray diffraction studies, was then examined by Raman spectroscopy and the resulting Raman spectrum of D_2_Q_15_K_2_ (with the solvent background subtracted) is shown in Figure 3(i). The signature bands in the spectrum, assigned to the dominant structural motifs, are clearly distinguishable above the noise level. The small negative feature at ∼1380 cm^−1^ is from an artefact produced by the spectrometer’s CCD camera. In the amide I and III regions, from 1600–1700 and 1200–1340 cm^−1^ respectively [7], the dominant features correspond to marker bands for extended β-sheet structure. In particular, the amide III band at 1239 cm^−1^ originates mainly from C-C and C-N stretching of residues in β-sheets, while the amide I band at 1659 cm^−1^ originates from the carbonyl stretching mode. Raman marker bands for β-sheet in the amide I region are typically reported at ∼1690 cm^−1^ and a small shoulder at this position is also observed here. This is the first report of a strong β-sheet Raman marker band at 1659 cm^−1^ that we are aware of, however Sharma *et al.* [25] reported an amide I marker band for β-sheet at the same frequency for a Q22 polyglutamine peptide in the infra-red spectrum. Therefore, this feature appears to be particularly sensitive to the identity of the residue composition of β-sheets and further indicates that the carbonyl groups within the glutamine side chains are contributing strongly to the corresponding vibrational mode. We also note that Raman marker bands for secondary structure elements are usually not as sharp as those observed here, which indicates that the β-sheet structure adopted by D_2_Q_15_K_2_ is particularly homogenous and rigid.

With a knowledge of the Raman spectrum for the fibrils of D_2_Q_15_K_2_ our attention turned towards the potential for the analysis of the protein structure *in vivo* using CARS microscopy. The spontaneous Raman cross-section is typically 10^−30^ [26] and for this reason Raman signals require long integrations times, meaning that spontaneous Raman is unsuitable for imaging dynamic living biological systems. However, imaging by CARS microscopy can be achieved at video-rates. [2] For CARS spectroscopy, as distinct from spontaneous Raman spectroscopy, resonant contributions from different vibrational modes and non-resonant background additively contribute to the complex spectral shape. In order to relate the Raman spectral data shown in figure 3(i) to that which would be expected from CARS microscopy, it was necessary to simulate spectra with various levels of non-resonant background present. The reported method of Bonn *et al*. [27] was established for the extraction of Raman line-shapes from CARS spectra. Here a reverse procedure is followed to reveal the potential CARS spectra from the experimental Raman spectrum for the fibrils. The results, shown in figure 3(ii), indicate that the amide I peak, seen in the Raman spectrum at 1659 cm^−1^, remains strong but is only slightly shifted to lower wavenumber in the CARS spectrum (50% non-resonant background contribution); see Figure S2 for spectra with 0%, 10%, 20% and 50% non-resonant background contribution. The peak seen at 1690 cm^−1^ in the Raman spectrum is less easy to observe in the predicted CARS spectra with a 50% non-resonant background contribution (Figure 3(ii)). The amide III band (detected in the Raman spectrum at 1239 cm^−1^) is both (i) shifted to lower wavenumber and (ii) weaker as a function of non-resonant contributions in the predicted CARS spectra (Figure 3(ii) and Figure S2). In order to confirm this approach, a Raman and CARS spectra of the fibrils of D_2_Q_15_K_2_ in water were recorded. The measured CARS spectrum (Figure S3) is found to be consistent with the predicted CARS spectral data (Figure 3 (ii) and Figure S2); the amide I marker band is found to be strong and marginally shifted to lower wavenumber and the amide III band is both shifted to lower wavenumber and weaker.

With a knowledge of the potential CARS spectrum, it is possible to establish the optimal vibrational frequency for imaging using our single frequency CARS microscopy method where the aim is to evaluate the structure of the aggregates of polyglutamine *in vivo*. This is where there is (i) a signature band for the protein structure and (ii) where non-resonant contributions have limited effect on the peak intensity and shift; it is for this reason that the amide I peak (seen in the Raman spectrum at 1659 cm^−1^) is that chosen for the following studies. It should be noted that the concentration of polyQ within an aggregate is anticipated to be higher than almost any other protein or molecule in the surrounding tissue (‘the background’), thus if a β-sheet structure of polyQ, as seen *in vitro*, is also present *in vivo*, then a CARS image obtained at ∼1657 cm^−1^ is anticipated to be optimal. Recent progress in stimulated Raman scattering, [28] a technique similar to CARS but with a closer relation to the spontaneous Raman spectra, and background-free CARS [29] might provide alternative approaches.

Transgenic *C.elegans* strains expressing Q40-YFP fusion proteins, as previously reported for other studies [13], [17], were then evaluated by CARS methods here. In this strain the chimeric protein Q40-YFP is expressed in the body wall muscle cells. As previously described in detail by Morimoto *et al*., *C. elegans* strains expressing Q40-YFP are shown to contain both polymorphic distribution of the chimeric protein Q40-YFP structures identified as diffuse and focused fluorescence patterns. And as already elegantly demonstrated, with aging there is an accumulation of aggregates into foci from the diffuse structures and the timescale for formation of the polyQ aggregate foci correlates with the toxicity for the *C. elegans* strains expressing Q40-YFP. [13].

The advantage of imaging a transgenic organism containing a fusion protein of a fluorescent protein and a polyQ sequence is that by performing both fluorescence imaging concurrently with CARS imaging, it is possible to pin-point regions that contain the protein of interest. A comparison of the fluorescence intensity against the CARS intensity at specific wavenumbers allows for an evaluation of the heterogeneity of the protein structure. The Raman spectra for green fluorescent protein and the mutant, yellow fluorescent protein (YFP) have been studied by Tonge *et al*. [30]; the predominant bands are seen at ∼1560 cm^−1^ with a rather weak band at 1662 cm^−1^. Thus by selecting the strong signature bands identified in the Raman (and predicted CARS) spectra that of D_2_Q_15_K_2_, at 1690 cm^−1^ and ∼1655–1659 cm^−1^, images to establish the presence of the β-sheet structure of polyglutamine *in vivo* are obtained. The spectral resolution of the CARS microscope is sufficiently high (3 cm^−1^) (See Materials and Methods and reference [29]) to allow for collection of CARS images at specific vibrational bands.

**Figure 4:**
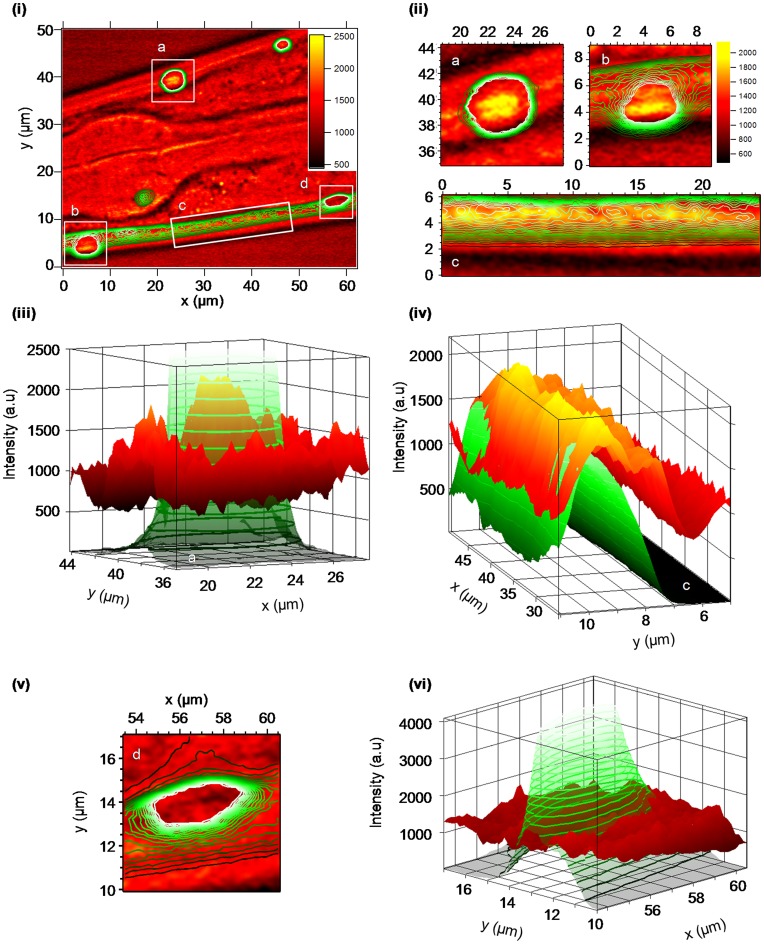
CARS and fluorescence microscopy images of the nematode, *C. elegans* (Q40-YFP) at 1 day old adult (day 4). (i) Image of the lateral view obtained by CARS microscopy (1657 cm^−1^); where the CARS intensity (in arbitrary units) is shown by the scale bar and the fluorescence intensity is shown by green contours. Four regions are identified (a,b,c,d) which are shown in an expanded form in figures (ii) to (vi) (ii) Expanded view of regions a, b, c from figure (i) where the CARS signal (1657 cm^−1^) intensity is greatest. The axes are in microns and correspond to the axes shown in figure (i), other than for region ‘c’ which is rotated; the axes are in microns. (iii) Plot of the region identified as ‘a’ in figures (i) and (ii) against intensity (in arbitrary units). The fluorescence signal is identified in green/green contours and the CARS signal (1657 cm^−1^) black-red-yellow with a scale as in figure (ii). (iv) Plot of the region identified as ‘c’ in figures (i) and (ii) against intensity (in arbitrary units). Note: the rotation, the cuticle and hypodermis of the *C. elegans* nematode is to the right hand side of the figure, rather than at the bottom of the figure as in (ii) expanded view ‘c’.

*C. elegans* strains expressing Q40-YFP were imaged by CARS microscopy from day 4 (day 1 adult) and older, at wavenumbers spanning the fingerprint region (1220–1750 cm^−1^) [7] and a small number imaged at 2850 cm^−1^ to detect lipids. [31] As already elegantly demonstrated by Morimoto *et al*., with aging there is an accumulation of aggregates from diffuse structures at approximately day 4 for *C. elegans* strains expressing Q40-YFP and this correlates with toxicity. [13] Shown in figure 4(i) is a CARS image obtained at 1657 cm^−1^ showing the lateral view of the pharyngeal region of the nematode, *C.elegans* at day 4 (Figure S4). The signal at ∼1657 cm^−1^ represents the most intense signals in the Amide I region in the predicted CARS spectra shown in figure 3(ii). Also shown are two-photon fluorescence images (as a contour plot) obtained concurrently. Similar CARS images were obtained at 1690 cm^−1^ (Figure S5) and other *C.elegans* nemotodes at day 4 and other regions were also imaged and found to contain similar CARS signals at this wavenumber. Of note, the CARS images at 2850 cm^−1^ (Figure S6) contain lipid droplets as reported by others using CARS methods [3], [32] as well as weaker signals for the regions of the image where the fluorescence signal is also co-located. As seen in figure 4(i), the internal body structure of the nematode has a significant effect on the CARS signal intensity at 1657 cm^−1^; structures within the metacorpus and isthmus (of the pharynx (see Figure S4)) and surrounding tissues can easily be observed. This is not surprising given the differences in refractive index for the tissues and body parts within the nematode, *C. elegans* [33], and the heterogeneity of other proteins with a CARS signal at 1657 cm^−1^. Within the image, shown in figure 4(i), both foci (labelled a, b, d in the figure) and diffuse (labelled c) Q40-YFP can clearly be identified by the fluorescence signal. The size of the ‘foci’ aggregates (a, b, d) is sufficiently big that large numbers of adjacent voxels represent the structure.

Shown in figure 4(ii) are expanded views of the identified regions (a, b, c) where the CARS signal at 1657 cm^−1^ is the most intense. The aggregate, labelled as ‘a’ in figures 4(i) and 4(ii) is shown as a 3D fluorescence and CARS intensity plot (figure 4(iii)). Even though the fluorescence signal is so intense that it has reached saturation, it is clear that the fluorescence and the CARS signals at 1657 cm^−1^ are co-located (See Figure S7 and Figure S8 for the CARS intensity profile (for data at 1690 cm^−1^ see Figure S9 and Figure S10))). The diffuse Q40-YFP labelled as ‘c’ in figures 4(i) and (ii) is shown as a 3D intensity plot in figure 4(iv). The fluorescence and the CARS signal intensities correlate well within the region identified as ‘c’ and thus indicate that both signals originate from the same protein, Q40-YFP. Figure 4(v) and (vi) show the expanded view and 3D intensity plot for an aggregate that is fluorescent, but shows no evidence for the CARS signal at 1657 cm^−1^ (or at 1690 cm^−1^ (Figure S5)). In fact the CARS signal at 1657 cm^−1^ is far weaker in the centre of the aggregate ‘d’ than in the surrounding regions (Figure S11 and Figure S12).

If the fluorescence is taken as a measure for the concentration of Q40-YFP then it would appear that the polyglutamine structure varies to such a degree that the β-sheet structure, indicated by the CARS signal at 1657 cm^−1^, is not present in aggregate ‘d’, and present only in the diffusely distributed regions ‘c’ and the centre of aggregates labelled as ‘a’ and ‘b’ in figure 4(i). Whilst it is possible that the Q40-YFP aggregate ‘d’ shown in figures 4(v) and (vi) may be so significantly dense that the signal is compromised in this region as a result of scattering of the excitation and emission light, it is difficult to explain why the β-sheet structure, detected by the CARS signal at 1657 cm^−1^ is observed in the centre of the aggregates in figure 4(ii) labelled ‘a’ and ‘b’ and that the fluorescence signal observed for aggregate ‘d’ is so intense. The aggregate ‘d’ shown in figures 4(v) and (vi) is located close to the diffuse pattern of Q40-YFP labelled as ‘c’ in figure 4(i). On closer examination the intensity of the CARS signal at 1657 cm^−1^ (or 1690 cm^−1^ (Figure S5 and Figure S13)), which is diffusely present within the muscle cells seen in the region ‘c’, is reduced at locations that approach the aggregate ‘d’ (Figure S11 and Figure S12). *C.elegans* (Q40-YFP) imaged at 5 days old or older, under similar conditions, show no evidence for a CARS signal at 1650–1700 cm^−1^ in the single densely aggregated Q40-YFP regions, identified by fluorescence; the diffusely located Q40-YFP is not observed at this time (Figure S14). It is therefore proposed that the Q40-YFP protein that is aggregated into foci is no longer β-sheet in structure.

As outlined previously by Morimoto *et al*., the influence of aging of the worms is correlated with the aggregation into foci of the chimeric fusion protein Q_n_-YFP and its toxicity, which is characterised by motility [13]. There it was found that the level of aggregation into foci of the Q40-YFP animals was found to increase up until day 4–5 and this correlated with the reduced motility measurements. Thus these current results suggest that there is a change in structure of the polyglutamine protein that correlates with the time point that the toxicity effects are observed. Cells are known to assemble misfolded and aggregation-prone proteins in intracellular, cytoplasmic structures such as inclusion bodies or “aggresomes” [34]. Misfolded protein species in inclusion bodies are made up of one particular protein that co-exists with various other proteins including heat shock proteins [35]. At present little is known about the formation of inclusion bodies, including polyQ aggregates. Knowledge of how the protein structure of polyQ aggregates evolve and change over time is critical for understanding their role in cell (dys)-function. In addition the impact of proteins such as chaperones or chemicals on the structure of polyQ *in vivo* remains little understood [36], [37], [38]. Our results suggest that the polyglutamine tracts *in vivo* assemble into cross β-sheet structures as found in fibres of the peptide, D_2_Q_15_K_2_ and htt exon 1 obtained *in vitro*. These structures migrate into foci, following which the polyQ cross β-sheet structure undergoes a metamorphosis into a different form. It is possible that this is a result of proteins, such as heat shock proteins, which co-assemble into the foci and disrupt the prevailing polyQ structure.

Previous fluororescence recovery after photobleaching (FRAP) studies on 4 day old *C. elegans* strains expressing Q40-YFP showed that the fluorescence of diffuse Q40-YFP structures recovered within 3 seconds and focal aggregates did not recover within 30 seconds, thus it had already been invoked by others that the diffuse and foci Q40-YFP structures exist as different biochemical states. [13] From the studies reported here we are able to state that the polyQ exists as a cross β-sheet structure as seen in the diffuse and early phase ‘foci’ aggregates, the protein structure of the more mature ‘foci’ aggregates in the *C. elegans* is different and not currently possible to identify.

This is the first time that CARS microscopy has been used for the evaluation of protein secondary structure *in vivo*. It is unfortunate that the protein structure of purified proteins, peptides and assemblies that have already been characterised *in vitro* by NMR or crystallography methods, have rarely been assessed by vibrational spectroscopy methods, such as Raman or infrared spectroscopy. The generation of libraries of Raman spectra of characterised proteins have the potential to enable CARS microscopy to become an invaluable tool for the identification of protein structure *in vivo*, particularly for misfolded proteins in protein aggregates that are a hallmark for age associated diseases.

## Materials and Methods

### Preparation of Fibrils of the Peptide, D_2_Q_15_K_2_ for Electron Microscopy, X-ray Fibre Diffraction and Raman Spectroscopy

The peptide D***_2_***Q***_15_***K***_2_*** was purchased from Pepceuticals Ltd, Nottingham, UK as a lyophilised powder at >95% purity. The peptide was dissolved in 2 micron filtered distilled water and allowed to assemble over three days at 5 mg/ml at room temperature.

### Electron Microscopy

4 µl of the peptide solution (assembled) was placed on 400 mesh, formvar/carbon coated copper grids (AGAR Scientific) and blotted using Whatman filter paper. The grid was then washed twice with filtered distilled water before staining using 2% w/v uranyl acetate in water and blotting. The grid was examined using a Hitachi7100 electron microscope operated at 80 kV and pictures were taken using a Gatan CCD camera.

### X-ray Fibre Diffraction

10 µl droplet of peptide solution (assembled) was placed between two waxed filled capillaries and allowed to air dry in a sealed environment. The partially aligned fibre sample was placed on a goniometer and X-ray fibre diffraction data was collected using a Rigaku rotating anode (CuKα) and RAxis IV++ detector. Exposure times were 15–20 minutes and the specimen to detector distance was set to 160 mm. Diffraction patterns were examined using CLEARER. [23].

### Raman Spectroscopy

The peptide solution (D_2_Q_15_K_2_, assembled) (5 mg/mL), as used for the electron microscopy and X-ray fibre diffraction studies, was pipetted into a quartz anti-reflection coated microfluorescence cell (Optiglass, U.K.) prior to Raman spectroscopy measurements. A ChiralRAMAN spectrometer (BioTools Inc., Jupiter FL, USA) operating in the backscattering mode, utilising a Nd:VO_4_ laser exciting at 532 nm (700 mW) and with a spectral resolution of 7 cm^−1^ was used to collect the Raman data. Using methods as previously reported for peptides and proteins [39], [40], the aqueous sample of fibrils was contained within a cuvette with an effective measurement volume of ∼5 mm^3^ and a total data collection time of 1 minute. Raman spectra were repeated over a 24 hour period and found to be consistent.

### *C. elegans* Samples

The *C. elegans* strain expressing the chimeric fusion of polyglutamine (Q40) to yellow fluorescent protein (YFP) as previously reported [13], was employed for these studies. Nematodes were handled by standard methods [41]. For imaging, animals were picked and anesthetized in a 10 µl droplet of 100 mM sodium azide on a microscope slide, before covering with a glass cover-slip, sealing and imaging immediately.

### CARS

The CARS setup is based on a Coherent Paladin Nd:YVO4 laser and an APE Levante Emerald optical parametric oscillator (OPO). The fundamental (1064 nm) of the laser is used as Stokes, whereas the signal from the OPO (tunable between 700–1000 nm) is used as the pump and probe. The beams are scanned over the sample by galvano mirrors (Olympus FluoView 300, IX71) and focused by a 20×0.5NA objective lens into the sample. Both beams have a power of several tens of milliwatts at the sample. The forward generated CARS signal is filtered from the pump and Stokes light and detected by a photomultiplier tube [42]. The simultaneously created two-photon fluorescence light is collected backward through the focusing objective lens and detected on a second photomultiplier tube. The lateral and axial resolution are 400 nm and 1 micrometer respectively. The spectral resolution is 3 cm^−1^ (5 ps pulses at 800 nm). All images are 512×512 pixels and obtained in 2 seconds.

## Supporting Information

Figure S1 **Transmission electron microscopy image of fibrils of D_2_Q_15_K_2_ that were used for the X-ray diffraction and Raman spectral studies.**
(TIF)

Figure S2 **Raman spectrum of the fibrils of D_2_Q_15_K_2_ (5 mg/ml) and predicted M-CARS spectra with various contributions of non-resonant background (0%, 10%, 20% and 50%).** (Data relates to figure 3 in the paper.)
(TIF)

Figure S3 **Raman and CARS spectra of fibril of D_2_Q_15_K_2_ (5 mg/ml) in water.** (a) CARS spectrum of fibril of D_2_Q_15_K_2_. (1200–3000 cm^−1^) and Raman spectrum (1200–1800 cm^−1^) (with the solvent background subtracted) (b) Expanded view of CARS spectrum of fibril of D_2_Q_15_K_2_. (1200–1800 cm^−1^). Samples were prepared for CARS spectra using 100 µl of the fibril containing aqueous solution (see Materials and Methods) which was added to a glass slide, dried slightly in air and covered with a cover slip and sealed with varnish. The spectral resolution is 7 cm^−1^ with 50 mW maximum average power on the sample, with an 80 MHz repetition rate and ∼5 ps pulses. The objective was a 1.2NA water immersion objective, the beam was set to scan at 25 us per pixel, and at the magnification used each pixel was a little more than one focal spot wide. The rest time between irradiation events at a single focal volume was ∼1 second, and imaging was never carried out in one sample region (∼100×100 microns) for more than 3 minutes continuously. All other CARS parameters are as detailed in the Materials and Methods section and reference 29.
(PDF)

Figure S4 **Schematic showing the pharyngeal region of the nematode,** ***C.elegans*** **in relation to images shown in** **figure 4** **.** (i) Image as for figure 4(i) as shown and labelled for the metacorpus and isthmus, and (ii) schematic drawing of the nematode, C.elegans (hermaphrodite) the pharyngeal region shown in the image of figure 4(i) is highlighted by the red rectangle.
(TIF)

Figure S5 **Image of the lateral view of the nematode,** ***C. elegans*** **(Q40-YFP) by CARS (1690 cm** ^−**1**^ **) and fluorescence microscopy.** This is a similar view as for the data shown in figure 4 (Note the data shown in figure 4 is for the CARS images obtained at 1657 cm^−1^). (scale bar for CARS signal intensity shown in left hand figure), fluorescence as green contours. There is a magnified image of aggregate ‘a’. A slight shift of the viewing regions between experiments means that the image acquired at 1690 cm^−1^ had to be shifted by x = 12 pixels = 2.2 µm and y = 2 pixels = 0.37 µm, therefore the region of the worm shown here is approximately (but not exactly) the same as in figure 4 for 1657 cm^−1^. Frames labelled ‘a’, ‘b’ and ‘c’ are those evaluated in figures S9, S10 and S13, respectively.
(TIF)

Figure S6 **Image of the lateral view of the nematode,** ***C. elegans*** **(Q40-YFP) obtained by CARS microscopy (2850 cm** ^−**1**^ **).** This is a similar view as for the data shown in figure 4 (Note the data shown in figure 4 is for the CARS images obtained at 1657 cm^−1^).
(TIF)

Figure S7 **Evaluation of the CARS (1657 cm** ^−**1**^ **) image of the nematode,** ***C. elegans*** **(Q40-YFP) labelled as aggregate ‘a’ (shown in** **figure 4(ii)** **and 4(iii)).** (i) CARS (1657 cm^−1^) intensity profile along muscle (with plot (iii) of intensity as a function of distance) (ii) CARS (1657 cm^−1^) intensity profile across the muscle (with plot (iv) of intensity as a function of distance). Figure (iii) and (iv) are intensity plot of CARS (1657 cm^−1^) signal as a function of distance (black), the intensity profile (averaged over 3 µm) is fitted with a Gaussian curve (red).
(TIF)

Figure S8 **Evaluation of the CARS (1657 cm** ^−**1**^ **) and fluorescence image of the nematode,** ***C. elegans*** **(Q40-YFP) labelled as aggregate ‘a’ in** **figure 4(i)** **.** Figure (i) shows the fluorescence (green contour plot) and the CARS (1657 cm^−1^) signals (black/grey). The red line follows the tissue structure in the muscle cells. In figure (ii) is a plot is the CARS signal intensity (a.u.) along the red line and the Gaussian fit.
(TIF)

Figure S9 **Evaluation of the CARS and fluorescence image (1690 cm** ^−**1**^ **) of the nematode,** ***C. elegans*** **(Q40-YFP) labelled as aggregate ‘a’ in figure S5.** (i) CARS (1690 cm^−1^) (black/white scale) and fluorescence images (green contour) for aggregate ‘a’ and adjacent muscle. Red line follows tissue structure through centre of aggregate. (See figure S8 for CARS data at 1657 cm^−1^). Figure (ii) CARS intensity (a.u.) as a function of distance along the red line of upper figure (black line) and fit of the data (red line).
(TIF)

Figure S10 **Evaluation of the CARS and fluorescence image (1690 cm** ^−**1**^ **) of the nematode,** ***C. elegans*** **(Q40-YFP) for region ‘b’ in figure S5.** (i) CARS (1690 cm^−1^) (black/white scale) and fluorescence images (green contour) for region ‘b’. Figure (ii) shows the plot of CARS intensity (a.u.) as a function of distance (pixels) corresponding to the pixels followed by the blue and pink lines in figure (i).
(TIF)

Figure S11 **Evaluation of the CARS (1657** **cm** ^−**1**^ **) and fluorescence image of the nematode,** ***C. elegans*** **(Q40-YFP) in** **figure 4(i)** **as ‘c’ and ‘d’.** The figure (i) shows the fluorescence (green contour plot) and the CARS (1657 cm^−1^) signals (black/grey). The data evaluated in the lower plot is obtained from the maximal fluorescence indicated by the pink/red line in figure (i). The figure (ii) shows a plot of the intensity of the fluorescence (green) and the CARS signal (black) as a function of distance along maximal fluorescence (in pixels). A fit of the data is shown in red. The fluorescence signal intensities correlate until a point ∼1× the width of the aggregate on the RHS. The CARS signal at 1657 cm^−1^ is negative as compared to the adjacent LHS region.
(TIF)

Figure S12 **Evaluation of the CARS (1657** **cm** ^−**1**^ **) image of the nematode,** ***C. elegans*** **(Q40-YFP) shown in** **figure 4(i)** **as ‘c’ and ‘d’.** (i) The upper figure shows the fluorescence (green contour plot) and the CARS (1657 cm^−1^) signals (black/grey). The CARS data evaluated in the lower plot is obtained along the pink/red line in (i). The lower plot (ii) shows the intensity of the CARS signal (red) where the fluorescence is maximal (Figure S11 (i)) and the intensity of the CARS signal (black) along the edge of the YFP fluorescence signal (shown in this figure S12 (i) as the pink\red line). A fit of the CARS data is shown in red. The CARS signal intensity decreases until a point ∼1× the width of the aggregate on the RHS. The CARS signal at 1657 cm^−1^ within the aggregate (d in figure 4(i) is negative as compared to the adjacent region (‘background’) identified by the red line.
(TIF)

Figure S13 **Evaluation of the intensity of the CARS (1690 cm** ^−**1**^ **) signals in the image of the nematode,** ***C. elegans*** **(Q40-YFP) in the region defined in figure S5 as ‘c’.** Figure (i) shows the fluorescence (green contour plot) and the CARS (1690 cm^−1^) signals (black/grey) for region ‘c’ in figure S5. The data evaluated in the figure (ii) is a plot of the CARS intensity versus pixel number (from left to right) following the maximal fluorescence indicated by the pink/red line in figure (i) and plotted in red/pink in figure (ii). In addition in figure (ii) the intensity of the CARS signal (black) obtained along the lowest intensity contour of the fluorescence. The CARS signal at 1690 cm^−1^ is negative as compared to the adjacent LHS region within the aggregate region.
(TIF)

Figure S14 **Images of the lateral view of the pharyngeal region of the nematode,** ***C. elegans*** **(Q40-YFP) at 2 day old adult (day 5) obtained by CARS and fluorescence microscopy (wavenumber identified in each image).** The fluorescence is identified in green. There is no evidence for a CARS signal in any of the images at the wavenumber identified).
(TIF)
