# Supplementary figures and images for: Polyglutamine Aggregate Structure In Vitro and In Vivo; New Avenues for Coherent Anti-Stokes Raman Scattering Microscopy
Source: PLoS One. 2012 Jul 20;7(7):e40536. doi: 10.1371/journal.pone.0040536 (PMC3401212; doi:10.1371/journal.pone.0040536)

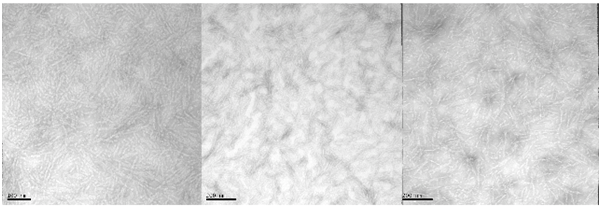

Supplement: Figure S1 — Transmission electron microscopy image of fibrils of D2Q15K2 that were used for the X-ray diffraction and Raman spectral studies. (TIF) [file pone.0040536.s001.tif]

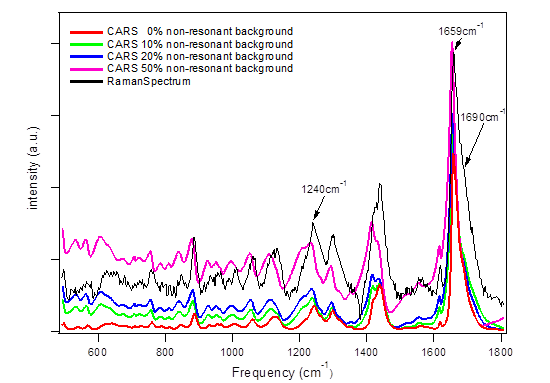

Supplement: Figure S2 — Raman spectrum of the fibrils of D2Q15K2 (5 mg/ml) and predicted M-CARS spectra with various contributions of non-resonant background (0%, 10%, 20% and 50%). (Data relates to figure 3 in the paper.) (TIF) [file pone.0040536.s002.tif]

(a)

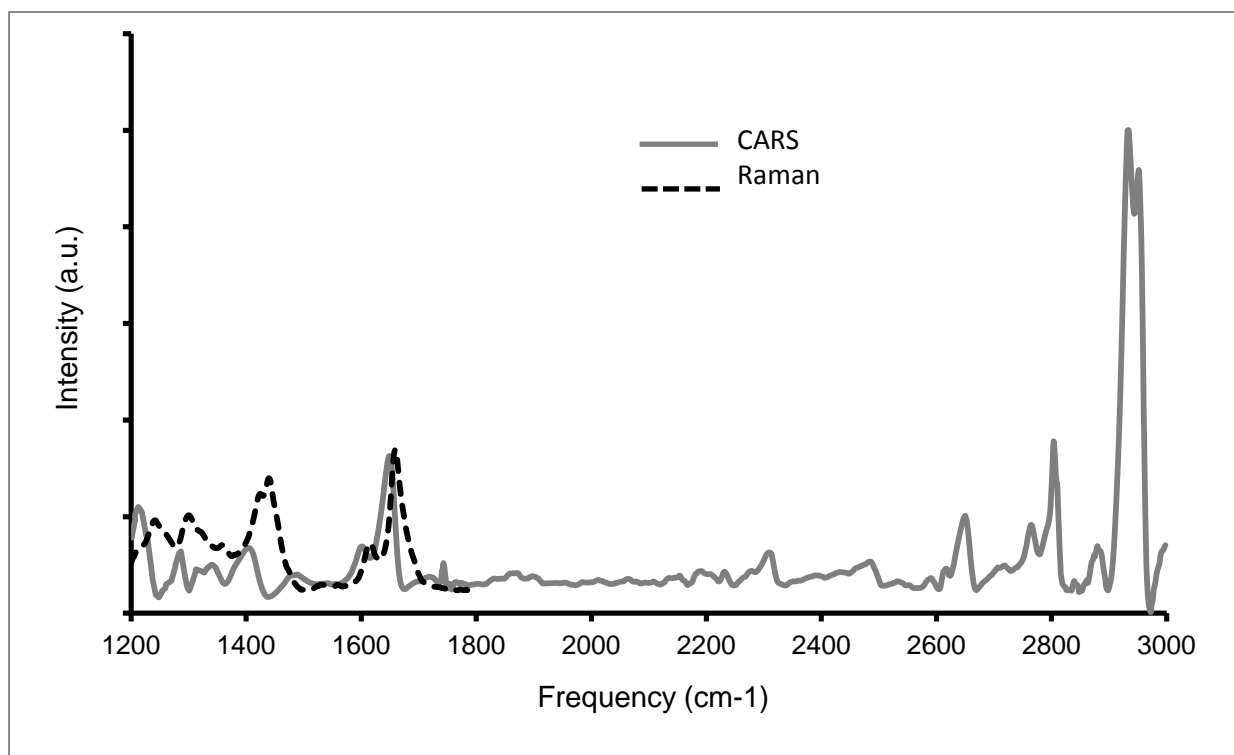

(b)

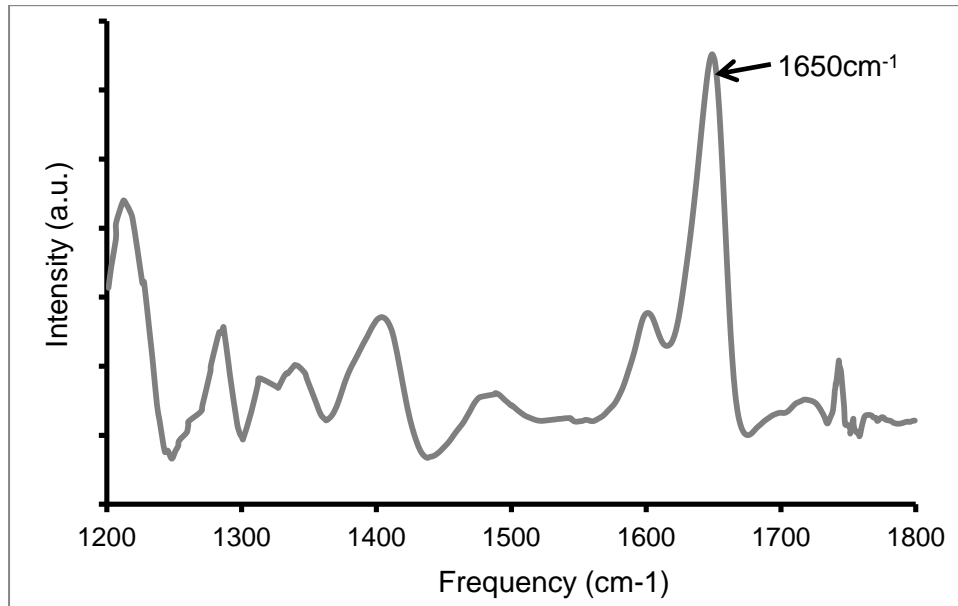

Supplement: Figure S3 — Raman and CARS spectra of fibril of D2Q15K2 (5 mg/ml) in water. (a) CARS spectrum of fibril of D2Q15K2. (1200–3000 cm−1) and Raman spectrum (1200–1800 cm−1) (with the solvent background subtracted) (b) Expanded view of CARS spectrum of fibril of D2Q15K2. (1200–1800 cm−1). Samples were prepared for CARS spectra using 100 µl of the fibril containing aqueous solution (see Materials and Methods) which was added to a glass slide, dried slightly in air and covered with a cover slip and sealed with varnish. The spectral resolution is 7 cm−1 with 50 mW maximum average power on the sample, with an 80 MHz repetition rate and ∼5 ps pulses. The objective was a 1.2NA water immersion objective, the beam was set to scan at 25 us per pixel, and at the magnification used each pixel was a little more than one focal spot wide. The rest time between irradiation events at a single focal volume was ∼1 second, and imaging was never carried out in one sample region (∼100×100 microns) for more than 3 minutes continuously. All other CARS parameters are as detailed in the Materials and Methods section and reference 29. (PDF) [file pone.0040536.s003.pdf]

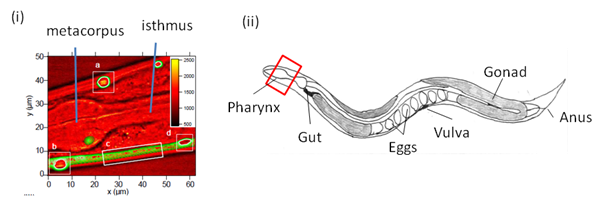

Supplement: Figure S4 — Schematic showing the pharyngeal region of the nematode, C.elegans in relation to images shown in figure 4 . (i) Image as for figure 4(i) as shown and labelled for the metacorpus and isthmus, and (ii) schematic drawing of the nematode, C.elegans (hermaphrodite) the pharyngeal region shown in the image of figure 4(i) is highlighted by the red rectangle. (TIF) [file pone.0040536.s004.tif]

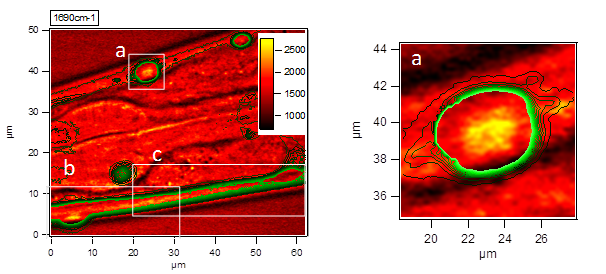

Supplement: Figure S5 — Image of the lateral view of the nematode, C. elegans (Q40-YFP) by CARS (1690 cm −1 ) and fluorescence microscopy. This is a similar view as for the data shown in figure 4 (Note the data shown in figure 4 is for the CARS images obtained at 1657 cm−1). (scale bar for CARS signal intensity shown in left hand figure), fluorescence as green contours. There is a magnified image of aggregate ‘a’. A slight shift of the viewing regions between experiments means that the image acquired at 1690 cm−1 had to be shifted by x = 12 pixels = 2.2 µm and y = 2 pixels = 0.37 µm, therefore the region of the worm shown here is approximately (but not exactly) the same as in figure 4 for 1657 cm−1. Frames labelled ‘a’, ‘b’ and ‘c’ are those evaluated in figures S9, S10 and S13, respectively. (TIF) [file pone.0040536.s005.tif]

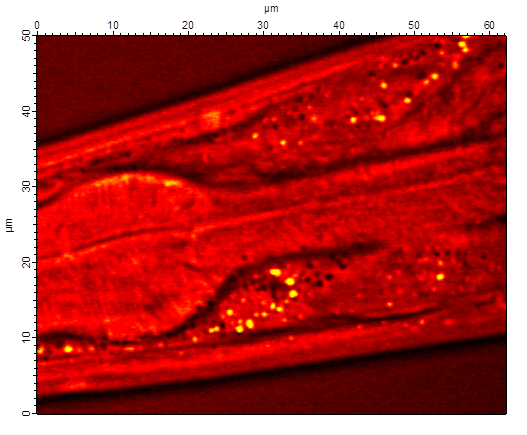

Supplement: Figure S6 — Image of the lateral view of the nematode, C. elegans (Q40-YFP) obtained by CARS microscopy (2850 cm −1 ). This is a similar view as for the data shown in figure 4 (Note the data shown in figure 4 is for the CARS images obtained at 1657 cm−1). (TIF) [file pone.0040536.s006.tif]

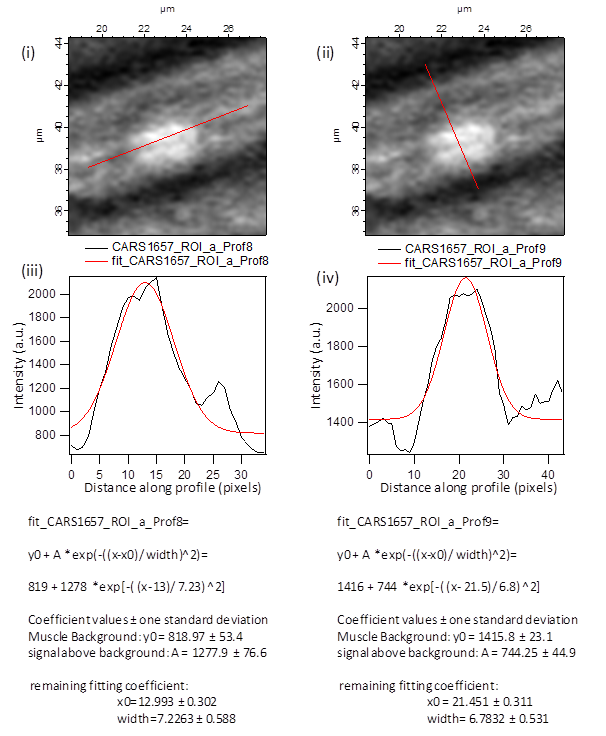

Supplement: Figure S7 — Evaluation of the CARS (1657 cm −1 ) image of the nematode, C. elegans (Q40-YFP) labelled as aggregate ‘a’ (shown in figure 4(ii) and 4(iii)). (i) CARS (1657 cm−1) intensity profile along muscle (with plot (iii) of intensity as a function of distance) (ii) CARS (1657 cm−1) intensity profile across the muscle (with plot (iv) of intensity as a function of distance). Figure (iii) and (iv) are intensity plot of CARS (1657 cm−1) signal as a function of distance (black), the intensity profile (averaged over 3 µm) is fitted with a Gaussian curve (red). (TIF) [file pone.0040536.s007.tif]

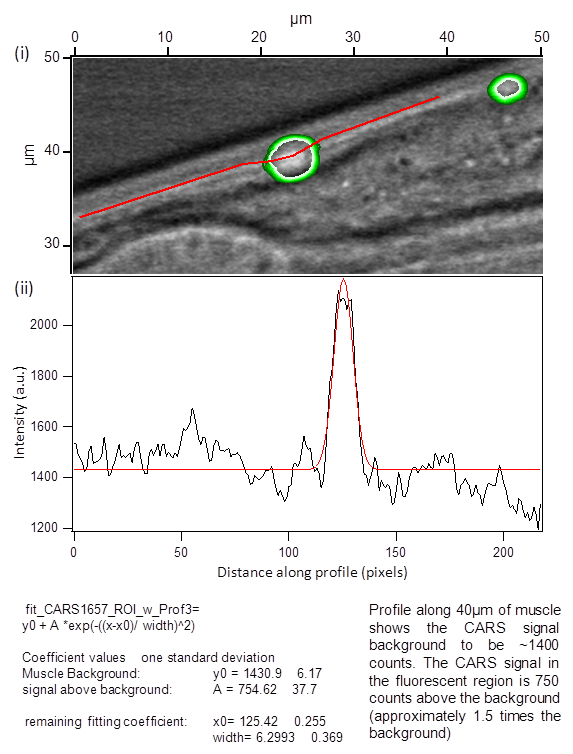

Supplement: Figure S8 — Evaluation of the CARS (1657 cm −1 ) and fluorescence image of the nematode, C. elegans (Q40-YFP) labelled as aggregate ‘a’ in figure 4(i) . Figure (i) shows the fluorescence (green contour plot) and the CARS (1657 cm−1) signals (black/grey). The red line follows the tissue structure in the muscle cells. In figure (ii) is a plot is the CARS signal intensity (a.u.) along the red line and the Gaussian fit. (TIF) [file pone.0040536.s008.tif]

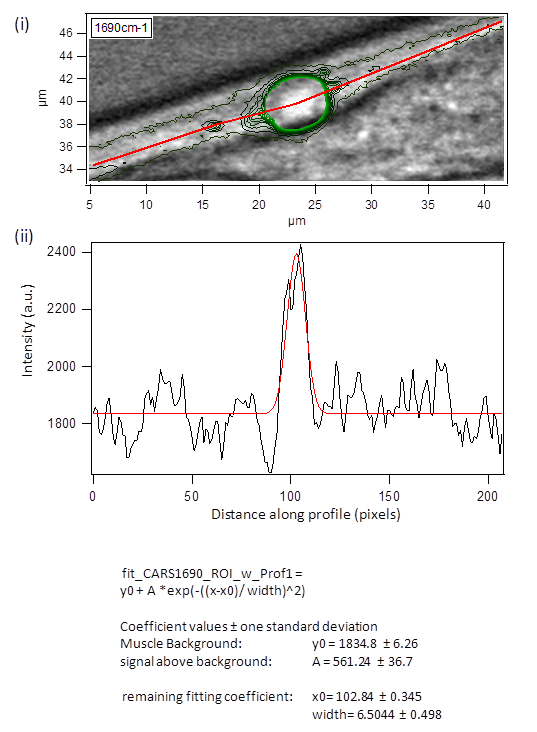

Supplement: Figure S9 — Evaluation of the CARS and fluorescence image (1690 cm −1 ) of the nematode, C. elegans (Q40-YFP) labelled as aggregate ‘a’ in figure S5. (i) CARS (1690 cm−1) (black/white scale) and fluorescence images (green contour) for aggregate ‘a’ and adjacent muscle. Red line follows tissue structure through centre of aggregate. (See figure S8 for CARS data at 1657 cm−1). Figure (ii) CARS intensity (a.u.) as a function of distance along the red line of upper figure (black line) and fit of the data (red line). (TIF) [file pone.0040536.s009.tif]

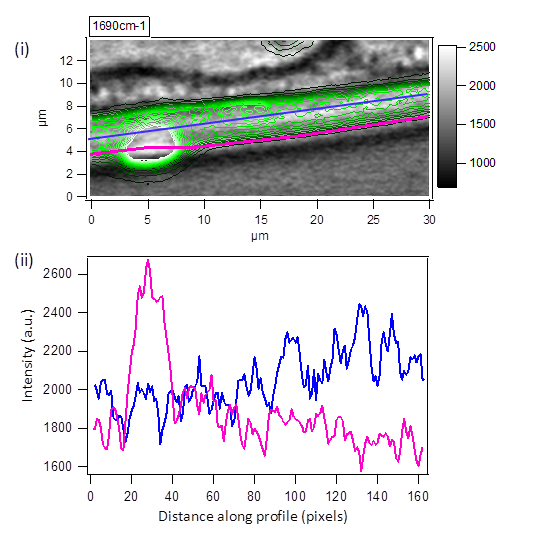

Supplement: Figure S10 — Evaluation of the CARS and fluorescence image (1690 cm −1 ) of the nematode, C. elegans (Q40-YFP) for region ‘b’ in figure S5. (i) CARS (1690 cm−1) (black/white scale) and fluorescence images (green contour) for region ‘b’. Figure (ii) shows the plot of CARS intensity (a.u.) as a function of distance (pixels) corresponding to the pixels followed by the blue and pink lines in figure (i). (TIF) [file pone.0040536.s010.tif]

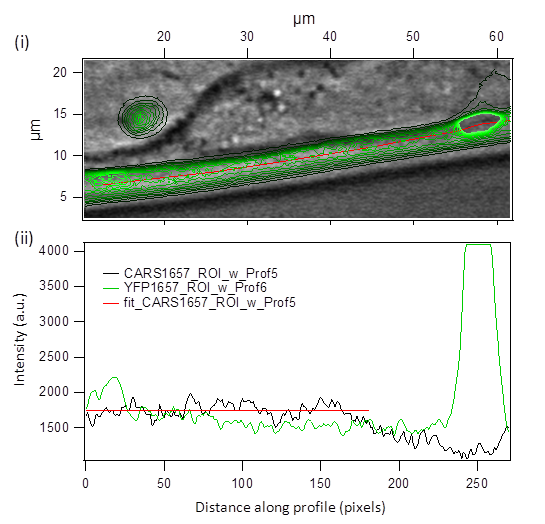

Supplement: Figure S11 — Evaluation of the CARS (1657 cm −1 ) and fluorescence image of the nematode, C. elegans (Q40-YFP) in figure 4(i) as ‘c’ and ‘d’. The figure (i) shows the fluorescence (green contour plot) and the CARS (1657 cm−1) signals (black/grey). The data evaluated in the lower plot is obtained from the maximal fluorescence indicated by the pink/red line in figure (i). The figure (ii) shows a plot of the intensity of the fluorescence (green) and the CARS signal (black) as a function of distance along maximal fluorescence (in pixels). A fit of the data is shown in red. The fluorescence signal intensities correlate until a point ∼1× the width of the aggregate on the RHS. The CARS signal at 1657 cm−1 is negative as compared to the adjacent LHS region. (TIF) [file pone.0040536.s011.tif]

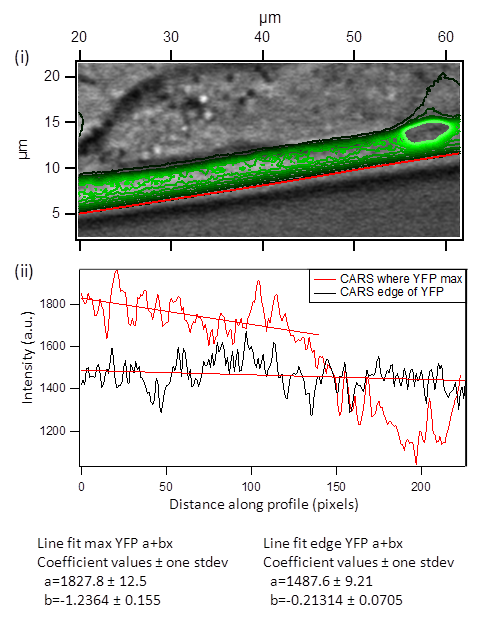

Supplement: Figure S12 — Evaluation of the CARS (1657 cm −1 ) image of the nematode, C. elegans (Q40-YFP) shown in figure 4(i) as ‘c’ and ‘d’. (i) The upper figure shows the fluorescence (green contour plot) and the CARS (1657 cm−1) signals (black/grey). The CARS data evaluated in the lower plot is obtained along the pink/red line in (i). The lower plot (ii) shows the intensity of the CARS signal (red) where the fluorescence is maximal (Figure S11 (i)) and the intensity of the CARS signal (black) along the edge of the YFP fluorescence signal (shown in this figure S12 (i) as the pink\red line). A fit of the CARS data is shown in red. The CARS signal intensity decreases until a point ∼1× the width of the aggregate on the RHS. The CARS signal at 1657 cm−1 within the aggregate (d in figure 4(i) is negative as compared to the adjacent region (‘background’) identified by the red line. (TIF) [file pone.0040536.s012.tif]

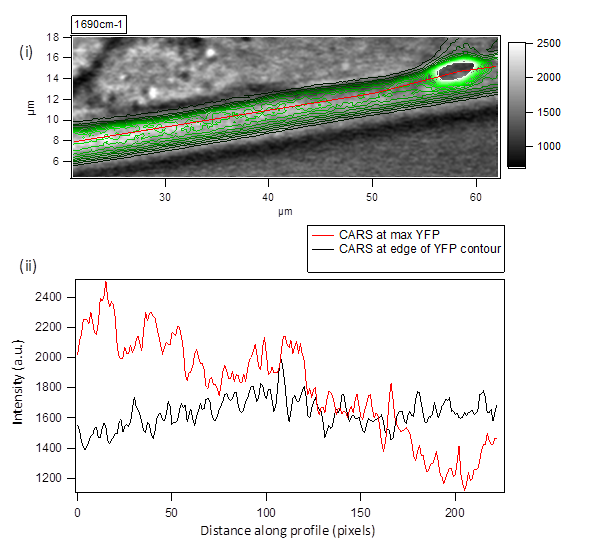

Supplement: Figure S13 — Evaluation of the intensity of the CARS (1690 cm −1 ) signals in the image of the nematode, C. elegans (Q40-YFP) in the region defined in figure S5 as ‘c’. Figure (i) shows the fluorescence (green contour plot) and the CARS (1690 cm−1) signals (black/grey) for region ‘c’ in figure S5. The data evaluated in the figure (ii) is a plot of the CARS intensity versus pixel number (from left to right) following the maximal fluorescence indicated by the pink/red line in figure (i) and plotted in red/pink in figure (ii). In addition in figure (ii) the intensity of the CARS signal (black) obtained along the lowest intensity contour of the fluorescence. The CARS signal at 1690 cm−1 is negative as compared to the adjacent LHS region within the aggregate region. (TIF) [file pone.0040536.s013.tif]

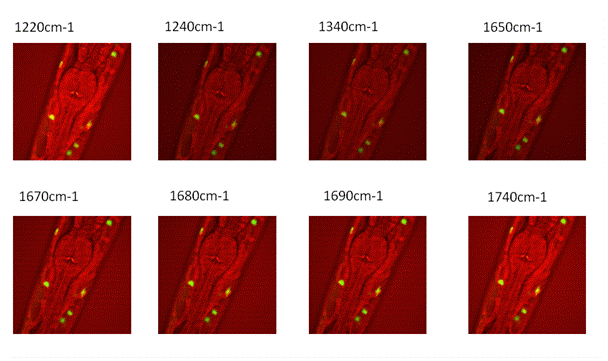

Supplement: Figure S14 — Images of the lateral view of the pharyngeal region of the nematode, C. elegans (Q40-YFP) at 2 day old adult (day 5) obtained by CARS and fluorescence microscopy (wavenumber identified in each image). The fluorescence is identified in green. There is no evidence for a CARS signal in any of the images at the wavenumber identified). (TIF) [file pone.0040536.s014.tif]
